# Efficient Genome Editing of *Magnetospirillum magneticum* AMB-1 by CRISPR-Cas9 System for Analyzing Magnetotactic Behavior

**DOI:** 10.3389/fmicb.2018.01569

**Published:** 2018-07-17

**Authors:** Haitao Chen, Sheng-Da Zhang, Linjie Chen, Yao Cai, Wei-Jia Zhang, Tao Song, Long-Fei Wu

**Affiliations:** ^1^Beijing Key Laboratory of Biological Electromagnetism, Institute of Electrical Engineering, Chinese Academy of Sciences, Beijing, China; ^2^University of Chinese Academy of Sciences, Beijing, China; ^3^France-China International Laboratory of Evolution and Development of Magnetotactic Multicellular Organisms, CNRS-Marseille/CAS, Beijing, China; ^4^Deep-Sea Microbial Cell Biology, Department of Deep Sea Sciences, Institute of Deep-Sea Science and Engineering, Chinese Academy of Sciences, Sanya, China; ^5^Key Laboratory of Earth and Planetary Physics, Institute of Geology and Geophysics, Chinese Academy of Sciences, Beijing, China; ^6^Aix Marseille Univ, Centre National de la Recherche Scientifique, LCB, Marseille, France

**Keywords:** CRISPR-Cas9, magnetotactic bacteria, magnetotaxis, Amb0994, dynamics simulation

## Abstract

Magnetotactic bacteria (MTB) are a diverse group of microorganisms capable of using geomagnetic fields for navigation. This magnetotactic behavior can help microorganisms move toward favorable habitats for optimal growth and reproduction. A comprehensive understanding of the magnetotactic mechanism at molecular levels requires highly efficient genomic editing tools, which remain underdeveloped in MTB. Here, we adapted an engineered CRISPR-Cas9 system for efficient inactivation of genes in a widely used MTB model strain, *Magnetospirillum magneticum* AMB-1. By combining a nuclease-deficient Cas9 (dCas9) and single-guide RNA (sgRNA), a CRISPR interference system was successfully developed to repress *amb0994* expression. Furthermore, we constructed an in-frame deletion mutant of *amb0994* by developing a CRISPR-Cas9 system. This mutant produces normal magnetosomes; however, its response to abrupt magnetic field reversals is faster than wild-type strain. This behavioral difference is probably a consequence of altered flagella function, as suggested with our dynamics simulation study by modeling *M. magneticum* AMB-1 cell as an ellipsoid. These data indicate that, Amb0994 is involved in the cellular response to magnetic torque changes via controlling flagella. In summary, this study, besides contributing to a better understanding of magnetotaxis mechanism, demonstrated the CRISPR-(d)Cas9 system as a useful genetic tool for efficient genome editing in MTB.

## Introduction

Magnetotactic bacteria (MTB) are a diverse group of prokaryotes that are capable of sensing and changing their orientation in accordance with geomagnetic fields, a behavior known as “magnetotaxis.” This behavior is thought to facilitate the dwelling of MTB within growth-favoring water columns or sediments with vertical chemical stratification (Blakemore, 1975; Faivre and Schüler, 2008; Lin et al., 2014; Chen et al., 2015). This unique capability is facilitated by special organelles that are intracellularly synthesized, membrane-enclosed ferromagnetic nanocrystals of magnetite (Fe_3_O_4_) and/or greigite (Fe_3_S_4_), called magnetosomes (Bazylinski et al., 1995; Barber-Zucker et al., 2016; Zhang et al., 2017a). Magnetosomes have emerged as a model for investigating prokaryotic organelle formation and biomineralization. Currently, genetic modification of MTB model strains relies on classical homologous recombination (HR) and transposon mutagenesis (Komeili et al., 2004; Jogler and Schüler, 2009; Wang et al., 2011; Komeili, 2012). However, detailed understanding of the molecular mechanism of magnetotaxis in MTB has been limited by these conventional approaches. Therefore, the ability to precisely manipulating MTB chromosome is highly desirable in applications ranging from genetic analysis of functional genomic loci to mechanisms of magnetotaxis.

Recently, clustered regulatory interspaced short palindromic repeats (CRISPR) and CRISPR-associated (Cas) proteins based genome editing systems have been developed (Barrangou et al., 2007; Brouns et al., 2008). The type II CRISPR-Cas9 from *Streptococcus pyogenes* consists of only two elements, an endonuclease Cas9 and engineered single-guide RNA (sgRNA) (Ran et al., 2013). Guided by a protospacer-adjacent motif (PAM) and a 20 nucleotide (nt) sequence matching the protospacer of the sgRNA, the Cas9-sgRNA ribonucleoprotein (RNP) complex binds specifically to the DNA target by sequence complementarity and induces DNA double-strand breaks (DSBs) (Jinek et al., 2012; Ran et al., 2013). A modified method, derived from the *S. pyogenes* CRISPR, CRISPR interference (CRISPRi) has been developed. An engineered nuclease-deficient Cas9, termed dCas9, enables the repurposing of the system for the targeted silencing of transcription (Larson et al., 2013; Qi et al., 2013; Dominguez et al., 2016; Shen et al., 2017). To date, CRISPR system has been successfully applied to a wide variety of eukaryotic organisms (Hwang et al., 2013; Li, 2015; Xu et al., 2015; Chen et al., 2017; Komor et al., 2017; Dong et al., 2018). The lethality of CRISPR-Cas9 system to bacterial strains has been reported (Gomaa et al., 2014; Cobb et al., 2015; Xu et al., 2015). To repurpose this system for genome engineering in prokaryotic cells, researchers use a homology repair donor DNA to efficiently generate mutations by CRISPR-Cas9 system, so that high-efficiency genomic deletions with or without selective marker can be performed via homology-directed repair (HDR) integration (Jiang et al., 2013). Subsequently, many groups adapted CRISPR-Cas9 system to various bacteria, such as *Escherichia coli* (Jiang et al., 2013), *Streptococcus pneumoniae* (Jiang et al., 2013), *Lactobacillus reuteri* (Oh and van Pijkeren, 2014), *Streptomyces species* (Cobb et al., 2015; Zeng et al., 2015; Hao et al., 2018), *Tatumella citrea* (Jiang et al., 2015), *Clostridium genus* (Wang et al., 2015b), *Bacillus thuringiensis* (Wang et al., 2017), and so on. Compared with HR, the advantage of the CRISPR-(d)Cas9 system is timesaving, because the counter-selection step of traditional HR based method is not required. Besides, CRISPRi could knockdown multiple genes at the same time, which is difficult to achieve with HR. *M. magneticum* AMB-1 serves as a model organism in MTB for studying biomineralization and magnetotaxis. Therefore, the development and application of CRISPR system in *M. magneticum* AMB-1 are highly demanded.

A large number of microorganisms fulfill their physiological needs through the active loco motions in their respective physicochemical environments. These bacterial motions, as responses to different extracellular stimuli, have been referred as various X-taxis behaviors, such as chemotaxis, phototaxis, aerotaxis, and magnetotaxis (Frankel et al., 2007; Bennet et al., 2014). In particular, magnetotaxis is found in MTB and is defined as the passive alignment of the cells to the geomagnetic field along with active swimming (Frankel and Bazylinski, 2009). A model has suggested that magnetotaxis, together with aerotaxis, enables MTB to move to suitable environmental conditions; this behavior is called “magneto-aerotaxis” (Bazylinski and Frankel, 2004; Zhang et al., 2014). However, several researchers think that magnetotactic behavior results from the active sensing of magnetic force to some degree (Greenberg et al., 2005; Pan et al., 2009). The active sensing process is a signal transduction system that depends on transmembrane chemoreceptors, known as methyl-accepting chemotaxis proteins (MCPs). These MCPs in turn could relay a signal to the flagellar motor switch protein, through a CheA-CheY signal transduction system (Falke and Hazelbauer, 2001; Philippe and Wu, 2010). Interestingly, *M. magneticum* AMB-1 contains a large number of MCP-encoding genes (Fukuda et al., 2006), among which *amb0994* plays a key role in magnetotaxis by interacting with MamK (Philippe and Wu, 2010; Draper et al., 2011). Overproduction of Amb0994 results in slow cellular response to changes along the direction of the magnetic field (Philippe and Wu, 2010). Moreover, Zhu et al. constructed a Δ*amb0994-0995* double-gene knockout mutant and recorded its response to magnetic field changes. Results suggested that *M. magneticum* AMB-1 cells use Amb0994-0995 to sense the torque and actively regulate the flagellar rotation bias accordingly to align its orientation with the external magnetic field (Zhu et al., 2014). However, construction of an *amb0994* single gene in-frame deletion mutant was unsuccessful. Direct evidence was thus missing to test the function of Amb0994 in the active response to magnetic field changes. Therefore, we purposed as a proof-of-concept to study the involvement of Amb0994 in magnetotaxis because this gene is involved only in magnetotactic behavior that is easy to observe.

In this study, we aim to develop an efficient genomic editing platform for *M. magneticum* AMB-1 by engineering CRISPR-Cas9 system. We successfully constructed *amb0994* knockdown (KD*amb0994*) and single-gene knockout strains (Δ*amb0994*). Analyses on the swimming behaviors of both mutants confirmed the function of Amb0994 in the active response of *M. magneticum* AMB-1 cells to magnetic field changes. Therefore, the new CRISPR system based genetic methods described here are useful references for facilitating versatile and efficient gene knockdown and/or knockout in MTB.

## Materials and methods

### Microorganisms and growth conditions

*E. coli* TOP10 was used for cloning and gene expression. WM3064 of *E. coli* was used as a donor strain in conjugations and grown in the presence of 300 μM diaminopimelic acid (DAP). A concentration of 50 μg/mL of kanamycin (Kan) or gentamycin (Gm) were used in the *E. coli* cultures. Cultures of wild-type (WT) and mutant strains of *M. magneticum* AMB-1 (American Type Culture Collection 700264) (Matsunaga et al., 1991, 2005) were grown microaerobically in modified enriched *Magnetospirillum* growth medium (EMSGM) containing per liter: 5 mL of Wolfe's mineral solution, 10 mL of Wolfe's vitamin supplement, 5 mL of 0.01 M ferric quinate, 0.68 g of potassium phosphate, 0.12 g of sodium nitrate, 0.74 g of succinic acid, 0.05 g of L-Cysteine-HCl, 0.2 g of polypeptone, and 0.1 g of yeast extract (Yang et al., 2001; Li et al., 2010). The pH was adjusted to 6.75, 7 g of agar was added per liter of EMSGM to prepare the agar plates, and the medium was autoclaved. Experiments were performed using a 100 mL Schott flask bottle or a 15 mL headspace (providing 10% air in the headspace for 2% oxygen) and incubated at 30°C. Generally, colonies appeared at the 6th−8th day after plating. In the *M. magneticum* AMB-1 cultures, kanamycin was added at 15 μg/mL to agar plates and 10 μg/mL to liquid cultures; gentamycin was added at 5 μg/mL.

### DNA manipulation, plasmid construction and conjugation

The enzymes for DNA modification were purchased from New England Biolabs (NEB) and Takara Biomedical Technology. Q5 High-Fidelity Polymerase (NEB, United States) was used for PCR amplifications, except for colony PCRs, which were performed using PrimerSTAR HS DNA polymerase (Takara, Japan). All PCR products and plasmids were purified using Monarch DNA Gel Extraction Kit (NEB, United States) and MiniBEST Plasmid Purification Kit (Takara, Japan), respectively. All plasmids and primers used in the study are listed in Tables S1, S2.

#### CRISPRi-based *amb*0994 suppression

Plasmid pCRISPRi-sgRNA*luxA* carrying dCas9, driven by the aTc-inducible tet promoter, Kan^R^ was constructed as previously described (Yin et al., 2018). In *M. magneticum* AMB-1, the tet promoter was replaced with the strong tac promoter, under the control of an isopropyl-beta-d-thiogalactopyranoside (IPTG). The sgRNA is a 102-nt-long chimeric noncoding RNA, consisting of a 20-nt target-specific complementary region, a 42-nt dCas9-binding RNA structure, and a 40-nt transcription terminator derived from *S. pyogenes*. The sgRNA was designed to target at the nontemplate (NT) DNA strand of a gene, in order to block transcriptional elongation. By using software packages sgRNACas9 and Blast, we designed CRISPR sgRNA for *amb0994* gene knockdown and potential off-target cleavage sites were also evaluated (Xie et al., 2014). The sgRNA sequence was as follows: *amb0994*: 5′-GTAATATCGACCATGATTGG-3′. The sgRNA was changed using the primers sgRNA-*amb0994*-F and sgRNA-*amb0994*-R. The method was formulated by Larson (Larson et al., 2013). We successfully constructed plasmid pCRISPRi-sgRNA*amb0994* and control plasmid pCRISPRi-no sgRNA. All constructions were confirmed by PCR, digestion, and sequencing.

#### Generation of Δ*amb*0994 single gene deletion mutant

Two methods were used to create a deletion of *amb0994*. First, Δ*amb0994* was constructed by HR in one-step (Figure S1). An approximately 1.0 kb upstream and downstream region flanking of *amb0994*, and the gentamycin resistance cassette from pUCGm were amplified using primers (0994-Left arm-*XbaI*-*XmaI*-F1, 0994-Left arm-*BamHI*-R2, 0994-Right arm-*BamHI*-F3, 0994-Right arm-*SacII*-R4, Gm-F and 3_Gm-R, Table S2). Amplified DNA fragments were ligated into the pMD18-T cloning vector (Takara, Japan) and cut by appropriate restriction enzymes; subsequently, they were cloned into the suicide vector pUX19 to form pUXsuc0994 (Wang et al., 2015a). Second, Δ*amb0994* was constructed by CRISPR-Cas9 system. Plasmid pCRISPR-sgRNA*amb0994* was constructed based on plasmid pCRISPRi-sgRNA*amb0994* from the following fragments: promoter tac; Cas9 was synthesized by in-fusion PCR that was performed with four primers (Primers Cas9-*XhoI*-F1, Overlap Cas9-R2, Overlap Cas9-F3, and Cas9-*XhoI*-R4) for gene mutation at two loci in dCas9; sgRNA (*amb0994*) was synthesized by GeneWiz Inc.; similarly, approximately 2.0 kb HDR DNA fragment, including the 1.0 kb left and right arms of editing template, was amplified from purified genomic DNA of *M. magneticum* AMB-1, which was also inserted with a gentamycin resistance cassette as same as HR method. Correct plasmid assembly was confirmed by PCR, digestion, and sequencing (Sangon biotech, China).

#### Complementation of Δ*amb*0994

Plasmid pAK0994 was assembled to complement Δ*amb0994*, named Com*amb0994*, a pBBR1MCS-based plasmid carrying a kanamycin resistance gene and expressing the *amb0994-gfp* fusion from a tac promoter. The method was performed as described previously (Komeili et al., 2006). *amb0994* was PCR amplified from *M. magneticum* AMB-1 with the primers Com*amb0994*-*EcoRI*-*gfp*-F and Com*amb0994*-*BamHI*-*gfp*-R. Amplified DNA fragments were digested by *EcoRI* and *BamHI* and ligated into plasmid pAK20 to create pAK0994. Relative abundance of Amb0994 in cells was checked with a fluorescence microscope. Statistical results showed that approximately 40% of cells did not express the protein (data not shown). This phenomenon might be due to the stability of the plasmid, the amount of expression from the tac promoter, or heterogeneous expression of *amb0994-gfp* from pBBR1MCS vector (Komeili et al., 2006). In addition, in order to exclude the effect of heterogeneous expression of *gfp* on trans-complementation, we constructed another plasmid without *gfp* (using primers Com*amb0994*-*xbaI*-F and Com*amb0994*-*KpnI*-R). The result showed no significant difference in the motion behavior and their growth between Com*amb0994-gfp and* Com*amb0994*.

Both HR and CRISPR conjugation experiments were performed as described previously with slight modification of the culture condition (Komeili et al., 2004; Philippe and Wu, 2010). WM3064 cells carrying each plasmid were grown at 37°C in LB medium, until an optical density (OD_600_) reached 0.5. As a donor, 500 μL of WM3064 culture was washed twice in LB medium and suspended with 200 μL LB medium with DAP. It was then mixed with 20 mL of exponentially growing *M. magneticum* AMB-1 culture at OD_600_ of 0.08 (containing 1 × 10^8^ cells). The donor-recipient cell suspension was concentrated by centrifugation into a final volume of 80 μL, and spot onto an EMSGM plate with DAP. The plate was incubated for 4 h at room temperature and the conjugations were suspended with 3 mL of EMSGM medium. Each of 100 μL conjugations was plated onto gentamycin and kanamycin-containing EMSGM plates for HR and CRISPR, respectively; then the plates were incubated in a microaerobic jar at 30°C for 6–8 days. The numbers of colonies obtained were used to calculate the conjugation efficiency taking account of the number of recipient cells initially used. Following conjugation, single colonies were grown and checked for the presence of the interference construct by PCR and Sanger sequencing. Resulting resistant strains were screened by PCR to determine the knockdown and knockout strains. This strain was checked for the absence of *amb0994*. The deletion region was sequenced to ensure that proper recombination occurred. In addition, six primers (p1, p2, p3, p4, p5, p6) were designed to verify the maintenance of magnetosome island (MAI) genes (Table S2). Six segments were chosen from *mamC, masA, mamB, mamY, mam O*′, and *mamK*. Clearance of the plasmid pCRISPR was performed on the plate with neither kanamycin nor DAP to confirm restoration of kanamycin sensitivity.

### Western blot analysis of *M. magneticum* AMB-1

*M. magneticum* AMB-1 colonies were transferred into 1.5 mL microcentrifuge tubes containing 1.5 mL EMSGM with 10 μg/mL kanamycin (for plasmid-bearing strains). A 1:10 dilution of *M. magneticum* AMB-1 cells in 1.5 mL of each culture was inoculated into 15 mL of EMSGM media with kanamycin. Cultures were sampled at the same growth stage and were run in technical replicates. Cultures were grown in approximately 2% O_2_ for 1 day; after incubation for 24 h, 0.5 mM IPTG was added into half of the cultures to induce expression of the genes encoded by the plasmids and incubated at 30°C for the next 24 h. Finally, cells were harvested by centrifugation at 10,000 rpm for 8 min at low temperature of 4°C for Western blotting analysis.

The procedure for Western blotting was based on a general protocol as previously described (Abreu et al., 2014). Pelleted cells were suspended in 20 mM Tris-HCl buffer (pH 7.4) and 5x protein-dyed buffer. All samples contained roughly equal amounts of cell by measuring cell density. Cells were heated at 100°C for 10 min and loaded onto a 7.5% polyacrylamide gel, which was first run at 80 V for 0.5 h, and then at 100 V for 1 h. Proteins were transferred onto a 0.22 μm polyvinylidene difluoride (PVDF) membrane at 100 V for 80 min. After the membrane was blocked for 1 h at room temperature with 5% milk-TBST (Tris-buffered saline-Tween 20), primary antibody (CRISPR-Cas9 monoclonal antibody recognizes both Cas9 and dCas9, Epigentek, United States) was applied (1:500) for 1 h at room temperature. The PVDF membrane was rinsed thrice for 10 min with TBST, and secondary antibody (Goat anti-mouse conjugated with horseradish peroxidase [HRP], Thermo Fisher Scientific, United States) (1:3,000) was applied for 1 h at room temperature. After washing with TBST thrice, antibody–protein complexes were then detected using Pierce CN/DAB substrate kit (Thermo Fisher Scientific, United States), according to the manufacturer's instructions.

### Transmission electron microscopy (TEM)

For TEM observations, *M. magneticum* AMB-1 cells were first suspended in water, drop-cast, and dried onto carbon-coated copper grids. All the samples were performed on a JEM-2100 TEM with an accelerating voltage of 200 kV, equipped with an Oxford SDD detector (X-MaxN80T). For each experiment, at least 400 magnetosome crystals and 80 cells were imaged. Based on TEM images, cells and crystal size were measured in the software ImageJ 1.51i. The method for analyzing the lengths of spirilla cells was to use polyline segments to fit the curve shape of the spirilla and measure the sum of the lengths of segments; and the widths were the diameters of cross-sections perpendicular to the cellular long axis. Shape factor was defined as width/length.

### CRISPRi-based regulation of gene expression analysis

A total of 50 mL of each culture was centrifuged (8,000 × *g*, 10 min, 4°C) and cell pellets were resuspended by vortexing in 1 mL of a mix of RNA protect solution and fresh culture medium (2:1). After 5 min incubation at room temperature, the cells were pelleted at maximum speed and rapidly frozen in liquid nitrogen for storage at −80°C. Total RNA was extracted from cellular pellets with the RNAeasy Plus Minikit (Qiagen, Germany), which was quantified by spectrophotometry (Implen NanoPhotometer, Germany). Contaminant DNA was removed from the RNA samples by digestion with gDNA Eraser. cDNA was produced from the RNA template by reverse transcription using the PrimeScript™RT reagent Kit with gDNA Eraser according to the manufacturer's instructions (Takara, Japan), and stored at −80°C for the next experiment. Reference genes are used to eliminate sample-to-sample variation. The RNA polymerase sigma factor *rpoD* was selected as a reference to normalize the data (Abreu et al., 2014). Primers are listed in Table S2, as designed by Primer 6 software. RT-qPCR was performed by following the manufacturer's instructions for a SYBR green real-time PCR mix using an ABI StepOne Real-time Detection System (Applied Biosystems, United States). The $2^{-\text{Δ}\Delta C_{\text{T}}}$ method was useful in the analysis of gene expression data (Livak and Schmittgen, 2001). All the samples from three independent experiments (biological replicates) were analyzed in triplicate (technical replicates), with negative controls included in each assay.

### Magnetospectrophotometry assay

Magnetospectrophotometry system was composed of a spectrophotometer (Hitachi U-2800, Japan) and an electromagnetic system (Zhao et al., 2007). OD and magnetic response (Cmag) of exponentially growing cultures were measured photometrically at 600 nm under the 4.5 mT homogeneous magnetic field, as described previously (Schüler et al., 1995). To avoid movement in the detection, 10 μm *m*-chlorophenylhydrazone (CCCP), a chemical inhibitor of oxidative phosphorylation, was added into the tube to stop cells from moving.

### Motility analysis of *M. magneticum* AMB-1 by experiment

*M. magneticum* AMB-1 motility behavior was analyzed using a microscope equipped with custom-made electromagnetic coils (Wang et al., 2016). Cells were subjected to a magnetic torque that reversed the direction of movement, resulting in an approximate U-trajectory after following field reversals, defined as “U turn” (Esquivel and De barros, 1986). Swimming velocities, diameters, and times of “U turn” were determined using the MTrackJ plugin for ImageJ (Meijering et al., 2012; Murat et al., 2015; Zhang et al., 2017b). The cells were placed on glass slides or in μ-Slide microchambers (Ibidi, Germany). To observe the glass slide, 4 μL of cells was observed under upright Olympus microscope using a long working distance 40x objective and recorded at 33 fps using a sCMOS camera (AndorNeo, England). Otherwise, 75 μL of cells suspension was placed in a microchamber and observed with a 40x phase-contrast microscope (Olympus, Japan), recorded at 20 fps using a Canon camera. In addition, the magnetic field was 1 mT in the “U turn” experiment without shielding the geomagnetic field. A total of ~300 traces of ~80 bacteria were analyzed in the “U turn” experiment, but ~30 bacteria were analyzed in alpha angle trajectory.

### Motility analysis of *M. magneticum* AMB-1 by simulation

We simulated the motion trajectory of bacteria in a magnetic field using an ellipsoidal model. The simulation was based on the concept that the moving *M. magneticum* AMB-1 cells, which are similar to ellipsoid, would suffer resistance in the fluid. The orientation of MTB under a magnetic field could be described by a partial differential equation:

(1) $$\begin{array}{r} I\frac{d^{2}\alpha }{dt^{2}}=PB\sin\alpha -C_{M}\frac{d\alpha }{dt} \end{array}$$

where *P* is the magnetic moment of one *M. magneticum* AMB-1, *B* is the applied magnetic field, and α is the angle between the direction of the magnetic field and the magnetic dipole moment. For *M. magneticum* AMB-1, the direction of magnetic dipole moment was the same as the major axis of the cell body, thus α could be measured by the angle between the instantaneous velocity vector and magnetic field line.

Other variables were defined as the following:

(2) $$\begin{array}{rcl} I & = & \frac{1}{5}m\left(a^{2}+b^{2}\right) \end{array}$$

(3) $$\begin{array}{rcl} C_{M} & = & 8\pi \mu ab^{2}\frac{\frac{4}{3}e_{0}^{3}\left[\left(2-e_{0}^{2}\right)/\left(1-e_{0}^{2}\right)\right]}{-2e_{0}+\left(1+e_{0}^{2}\right)\ln\;\left[\left(1+e_{0}\right)/\left(1-e_{0}\right)\right]} \end{array}$$

where *I* is the product of inertia of *M. magneticum* AMB-1, *m* is the mass, and *a* and *b* are the halves of the length and width of a *M. magneticum* AMB-1 cell, respectively. $C_{M}\frac{d\alpha }{dt}$represents the resistance torque applied to the *M. magneticum* AMB-1 cell. *C*_*M*_ is the coefficient of the resistance torque. In the expression (3) of *C*_*M*_, μ is the viscosity coefficient of the culture medium, and *e*_0_ is the auxiliary variable, which is defined as $e_{0}=\frac{1}{a}\sqrt{a^{2}-b^{2}}$.

Using the derivation method by Esquivel and De Barros, we can obtain the following expression of the alpha angle (Esquivel and De barros, 1986):

(4) $$\begin{array}{r} \alpha =2\mathrm{arc}tg\left(\sqrt{\frac{kT}{2PB}}\cdot e^{\frac{PB}{C_{M}}\cdot t}\right) \end{array}$$

where *k* is the Boltzmann's constant (1.38 × 10^−23^), *T* is the absolute temperature (K), and *t* is time.

Using the expression of (4), we calculated the orientation of *M. magneticum* AMB-1 under a magnetic field. If the bacterium has no flagella, or if the influence of flagellar movement and resistance in the fluid is not considered, the *C*_*M*_, defined as the expression of (3), could be utilized. If the bacterium has a flagellum, and flagellar influence suggesting a flagella movement signal provided by cells is considered, a modified *C*_*M*_ should be used. According to the research of Steinberger et al., when the *M. magneticum* AMB-1 is modeled as an ellipsoid connected with a wire fixed to one end of its body, the *C*_*M*_ should be multiplied by 4.67 (±0.47) (Steinberger et al., 1994). In our work, *C*_*M*_ was multiplied by 5 based on the expression of (3) for the case to consider the influence of flagellar movement and resistance in the fluid.

### Statistical analysis

All statistical analyses were performed using SPSS 22.0 (SPSS, IBM). Two-tailed Student's *t*-test was used in transcription level analysis. One-way analysis of variance was used to investigate the difference in velocities with magnetic field increase. Size and shape factor of cells and magnetosomes, number of magnetosomes in one cell were analyzed using Mann–Whitney *U*-test. Each experiment was repeated thrice, and all data are expressed as mean ± SD. Level of significance of the differences observed between the control and test samples were expressed as one or two stars, for ^*^P < 0.05 and ^**^P < 0.01. A *P-*value of < 0.05 was considered significant in all statistical tests.

## Results

### CRISPRi-based *amb0994* gene knockdown in *M. magneticum* AMB-1

**Figure 1:**
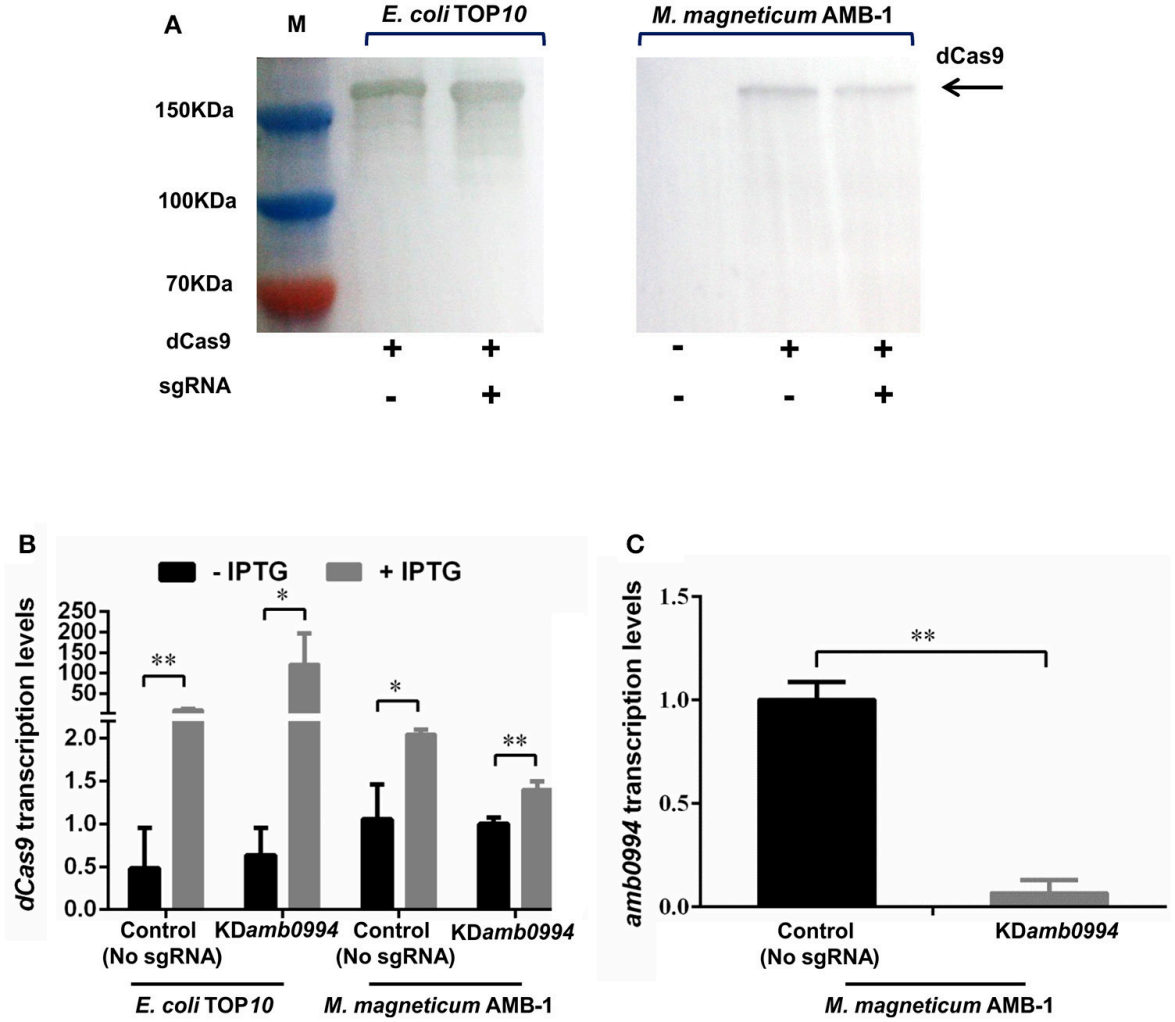
CRISPRi efficiently represses the *amb0994* gene transcription. **(A)** Western blot detection of dCas9 after SDS-PAGE with whole cell samples from experiment. The M represents the positions on the gel of the molecular mass markers (from top to bottom: 150, 100, and 70 kDa). **(B)** *dCas9* transcription levels of the control (plasmid without sgRNA) and test strains in *E. coli* and *M. magneticum* AMB-1 cells (*N* = 3 per strain) were determined. The error bars represent the standard deviation of samples. **(C)** The relative expression levels of *amb0994* in the KD*amb0994* compared to control strains without sgRNA. The levels of transcription were calculated relative to the housekeeping gene *rpoD* (*N* = 9 per strain). The error bars represent the standard deviation of samples. *P < 0.05, **P < 0.01.

CRISPRi system can be used to knockdown gene expression in eukaryotes and prokaryotes. To apply CRISPRi in *M. magneticum* AMB-1 cells, we first constructed a dCas9 gene repression plasmid. We chose to target the NT strand of *amb0994*, which should give more effective transcriptional repression as reported previously (Qi et al., 2013). The constructed plasmids were introduced into *M. magneticum* AMB-1 by conjugation as described in the “Materials and Methods” section. To first confirm if dCas9 is expressed in bacterial cells upon induction with IPTG, western blot was performed with dCas9/Cas9 antibodies and a band of 160 kDa was observed, corresponding to the anticipated molecular weight of dCas9 protein (Figure 1A). Further RT-qPCR analysis showed that the expression of dCas9 was significantly increased with IPTG induction (Figure 1B). IPTG was thus used in all experiments. In addition, as shown in Figures 1A,B, the expression levels of dCas9 upon IPTG induction are much lower in *M. magneticum* AMB-1 than in *E. coli* TOP10.

We analyzed the consequence of dCas9 production on the transcription level of *amb0994* (Figure 1C). The IPTG induced expression of a plasmid-borne dCas9 resulted in *amb0994* repression in cells expressing sgRNA targeting the NT strand of *amb0994*. The relative mRNA level of *amb0994* was reduced by 93% (*P* < 0.01) versus the control strain, wherein only dCas9 but not sgRNA was present. Therefore, the CRISPRi-dCas9 system could be used to efficiently knockdown the expression of *amb0994*.

### Efficient genome editing by CRISPR system as compared to HR

CRISPRi-dCas9 is an efficient approach for studying gene functions and for engineering genetic regulatory systems because of its sequence-specific control of gene expression on a genome-wide scale. However, off-target effects of CRISPRi technology might perturb gene expression at off-target sites. Previously reported CRISPR genetic modification technology has been successfully applied to a wide variety of organisms. To further verify the function of Amb0994 and assess the ability of Cas9-sgRNA assisted HDR in introducing a mutated locus in *M. magneticum* AMB-1 strain, we constructed an *amb0994* in-frame deletion mutant using either CRISPR or one-step HR method.

**Figure 2:**
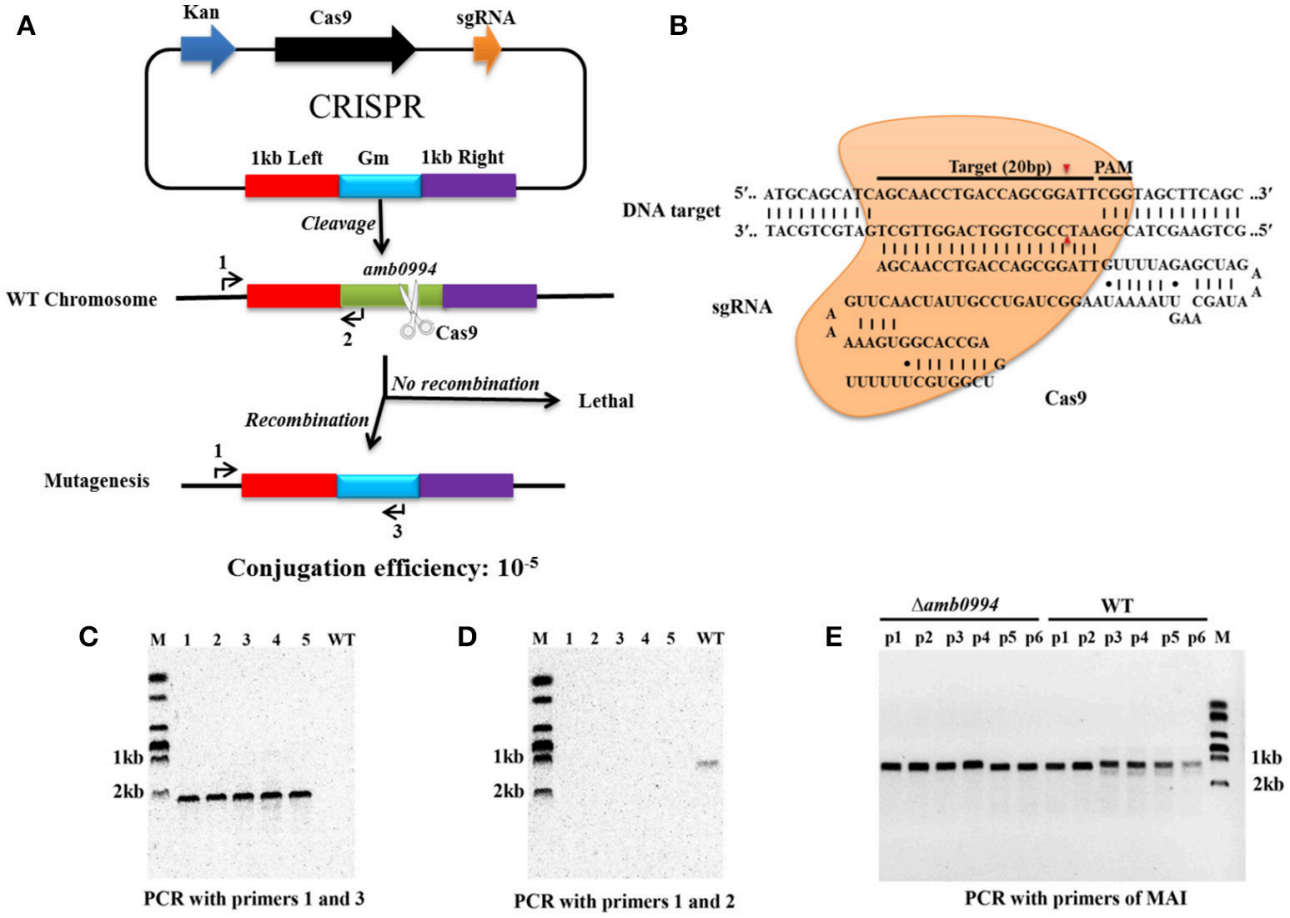
CRISPR-Cas9-assisted genome editing in *M. magneticum* AMB-1 cells. **(A)** Strategy for deletion of the *amb0994* gene by CRISPR-Cas9 assisted HDR in *M. magneticum* AMB-1 cells. An sgRNA transcripts guide Cas9 nuclease to introduce DSBs at ends of *amb0994* gene, while a codelivered editing template repairs the gap via HR. Kan is kanamycin. Gm is gentamycin. **(B)** Schematic of RNA-guided Cas9 nuclease uses for editing of the AMB-1 *amb0994*. An sgRNA consisting of 20 nt sequence (black bar) guide the Cas9 nuclease (orange) to target and cleavage the genomic DNA. Cleavage sites are indicated by red arrows for ~3 bp upstream of PAM. **(C,D)** PCR evaluation of *amb0994* deletion from five colonies (1–5) with WT control. **(E)** Six fragments within MAI were amplified to evaluate the maintenance of genomic MAI during deletion.

We performed CRISPR-Cas9-based gene knockout experiment. By designing a HDR donor template with homology arms near the Cas9-sgRNA, sgRNA transcripts could guide Cas9 nuclease to introduce DSBs at the ends of the *amb0994* gene, while a co-delivered editing template repairs the gap via HDR (Figures 2A,B). The conjugation efficiency was 10^−5^. Recombination event was evaluated by colony PCRs on genomic DNA with primers that anneal outside of homology arms and the marker region of gentamycin resistance cassette. Among 106 randomly selected colonies the recombination occurred in 90 conjugants. As shown in Figure 2C, a PCR amplification with primers annealing with upstream of 1.0 kb left arm (primer 1) and gentamycin resistance cassette (primer 3) is performed; An approximately 2.0 kb band indicating deletion of *amb0994* was observed in five representative colonies, whereas no band was observed when WT genomic DNA was used as the PCR template. The result showed that the gentamycin resistance cassette had replaced *amb0994*. In addition, the deleted region was test by PCR with primers 1 and 2. As expected, a 1.0 kb band was amplified with the WT genomic DNA, but not with genomic DNA of *amb0994* mutant strain (Figure 2D). The PCR fragment was sequenced with internal primers to determine whether the intended deletion was introduced or not. In the meantime, we designed a suicide plasmid of pUXsuc0994 for *amb0994* deletion through one-step HR as described in the “Materials and Methods” section. The conjugation efficiency was approximately 10^−6^ and the deletion of *amb0994* was obtained in 1 of 101 conjugants analyzed. The efficiencies of both conjugation and deletion were less compared with those of the CRISPR-Cas9-based knockout method. The low efficiencies might be partially due to the imposed one-step HR procedure, which was performed in order to compare with the one-step CRISPR-Cas9 method. The time-spans for the construction of *amb0994* deletion mutants by CRISPR-Cas9 method or one-step HR were both approximately 15–20 days. Two-step HR method is more efficient than one-step HR in deletion mutant construction, but it requires longer time-span. A schematic comparison between the one-step HR with insertion of a selective marker as used in this study for editing of *amb0994* and two-step HR (common route) is shown in Figure S1. In conclusion, we successfully used both CRISPR-Cas9 system and HR methods to knockout the *amb0994* gene in *M. magneticum* AMB-1.

In addition, MTB share a conserved genomic island, termed MAI, which is involved partly in magnetosome formation and encodes most of the proteins that are physically associated with purified magnetosomes (Fukuda et al., 2006). MAI is genetically unstable, which often results in frequent spontaneous loss of the magnetic phenotype (Bo et al., 2012). We designed six pairs of primers to evaluate the maintenance of genomic MAI during gene deletion. Results showed that MAI was not lost in Δ*amb0994* (Figure 2E). Taken together, all these results showed that the CRISPR-Cas9 system has higher editing efficiency compared with one-step HR for construction of *amb0994* deletion mutants.

### Effect of Amb0994 suppression or deletion on magnetosome formation in *M. magneticum* AMB-1

After constructing KD*amb0994* and Δ*amb0994*, we checked the magnetosome synthesis by analyzing the Cmag, size, shape factor and number of magnetosomes in KD*amb0994*, Δ*amb0994*, control, and Com*amb0994*. We also analyzed the length and shape factor of these cells. Given the inherent toxicity of overexpressed dCas9, we constructed a plasmid with dCas9 but not sgRNA and used it as negative control to KD*amb0994*, whereas the wild-type *M. magneticum* AMB-1 without plasmid was used as the control to Δ*amb0994* and Com*amb0994*.

**Figure 3:**
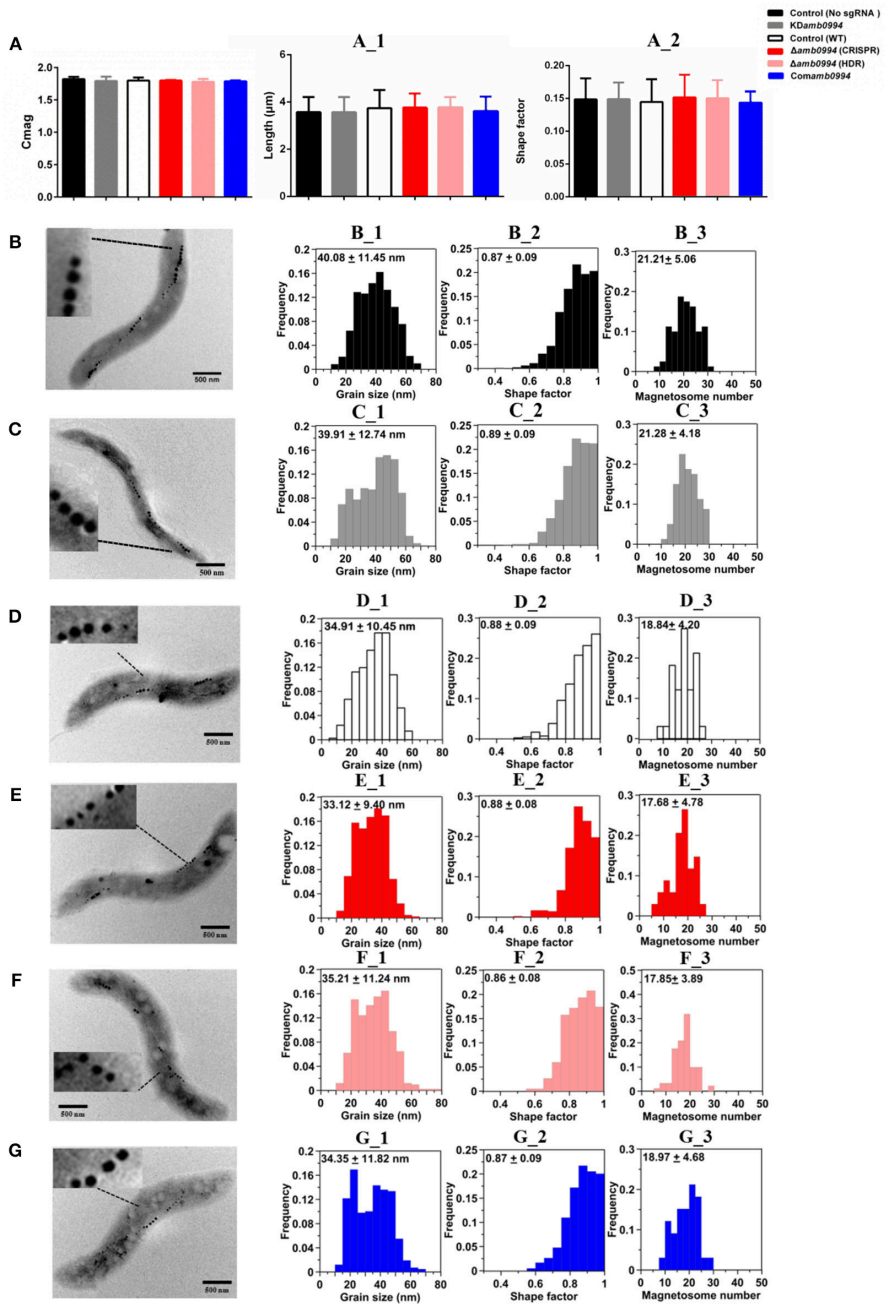
Magnetosome mineralization is not affected by CRISPR-based *amb0994* suppression or deletion in *M. magneticum* AMB-1. **(A)** Cmag curves of control, KD*amb0994*, Δ*amb0994* and Com*amb0994* strains under the 4.5 mT magnetic field, recording of AMB-1 treated with CCCP. (**A_1–2**) Cell size and shape factor of control, KD*amb0994*, Δ*amb0994*, and Com*amb0994* strains. TEM images of control strain without sgRNA **(B)**, KD*amb0994* **(C)**, WT control **(D)**, Δ*amb0994* by CRISPR **(E)**, Δ*amb0994* by HDR **(F)**, and Com*amb0994* **(G)**. **(B_1–3)–(G_1–3)** Histogram analyses of the number, size, and shape factors of magnetosomes in these strains. The scale bar is 500 nm.

As shown in Figure 3A, suppression or deletion of *amb0994* do not affect the value of Cmag versus the control. We analyzed the size, shape factor, and number of magnetosomes in *M. magneticum* AMB-1 cells according to TEM observations (Figures 3B–G). Result showed that the sizes of the magnetosome were 40.08 ± 11.45 nm and 39.91 ± 12.74 nm for the control (no sgRNA) and KD*amb0994*, respectively (Figures 3B_1,C_1). The shape factors were 0.87 ± 0.09 and 0.89 ± 0.09 (Figures 3B_2,C_2). The average numbers of magnetosomes in each cell were 21.33 ± 5.87 and 21.28 ± 4.18 (Figures 3B_3,C_3). Statistical analysis revealed insignificant differences between KD*amb0994* and negative control (no sgRNA) (*P* > 0.05). Moreover, we further analyzed the TEM data in Δ*amb0994*, Com*amb0994*, and WT strains. Indeed, the size, shape factor, and number of magnetosomes in these strains were the same as in the CRISPRi strains (Figures 3D_1–3,E_1–3,F_1–3, G_1–3). These results indicated that suppression or deletion of *amb0994* in *M. magneticum* AMB-1 would not interfere with the synthesis of magnetosomes. In addition, TEM analysis also revealed that the length and shape factor of cells of KD*amb0994*, Δ*amb0994*, control, and Com*amb0994* strains were statistically similar (Figure 3A_1–2).

### Amb0994 is involved in the response to the magnetic field

To further explore the role of Amb0994, we analyzed the motility behavior of *M. magneticum* AMB-1 in a laboratory magnetic field by an optical microscope. Studies have shown that the motion trajectory of MTB is normally a “U turn” when a reversal magnetic field is applied. The swimming velocities, diameters, and times of “U turn” following field reversals were measured as described in the “Materials and Methods” section. After 48 h of culture, the cells were placed in microchambers or glasses with an applied uniform magnetic field.

**Figure 4:**
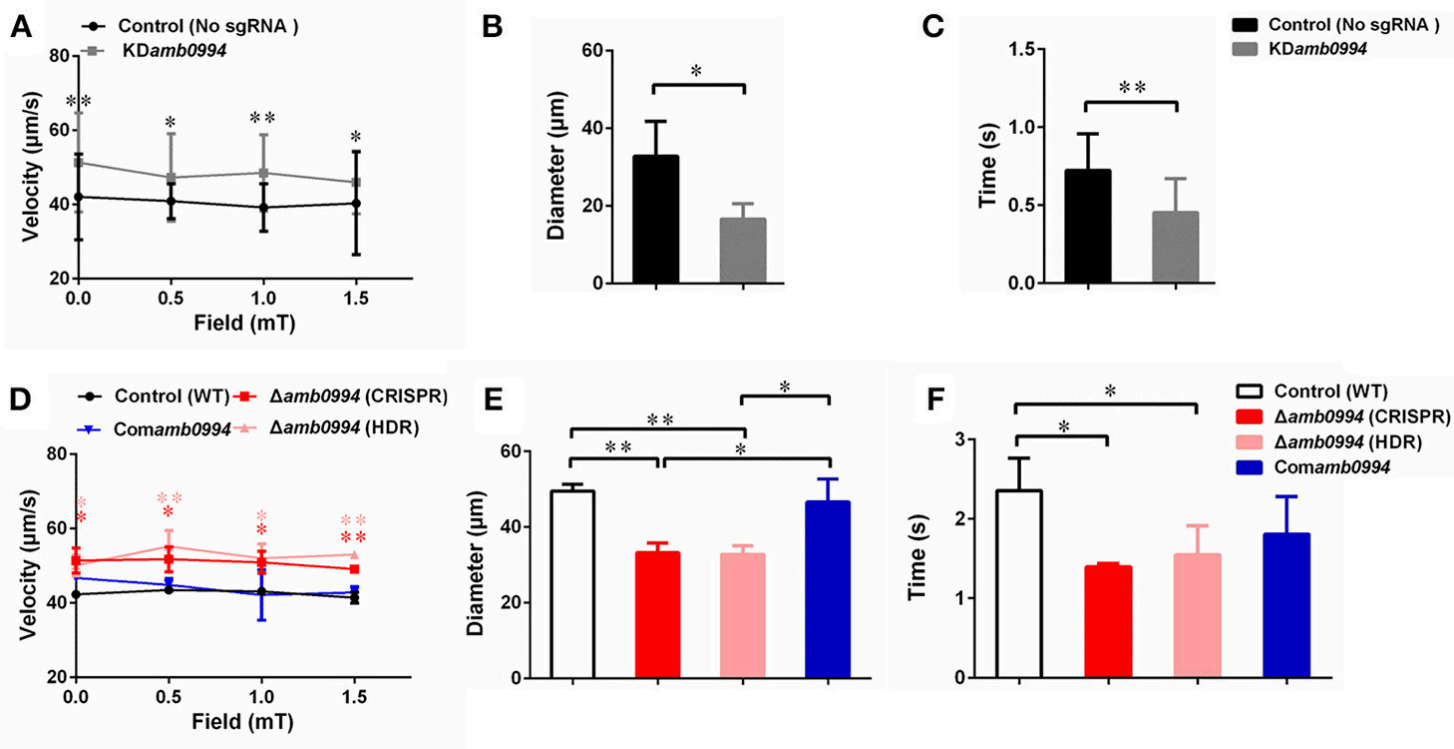
Effects of *amb0994* suppression or deletion on the swimming behaviors of *M. magneticum* AMB-1 cells. **(A,D)** Swimming velocities were measured under different fields. Red stars represent the statistical analyses performed between Δ*amb0994* (CRISPR) and WT control, whereas pink stars show statistical analyses between Δ*amb0994* (HDR) and WT control. **(B,C,E,F)** Diameters and times of “U turn” were analyzed in a 1 mT uniform magnetic field. Gray lines and bars represent the KD*amb0994* cells, black lines indicate control without sgRNA or wild-type groups, red and pink indicate Δ*amb0994*, and blue bars show Com*amb0994* strains. *P < 0.05, **P < 0.01.

Magnetic field strengths (0, 0.5, 1, and 1.5 mT) had no apparent effects on the velocities of KD*amb0994* cells and control strains, whereas the average swimming velocities of KD*amb0994* were significantly higher than those of the control (*P* < 0.05) (Figure 4A). More importantly, the diameters of “U turn” in the cells of KD*amb0994* under 1 mT magnetic field were smaller, and the times of “U turn” in KD*amb0994* were shorter compared with those of the control (Figures 4B,C, *P* < 0.05, Movies S1, S2). These results indicated that the knockdown mutant reacted faster and spent less time to achieve the “U turn” in response to magnetic field reversal.

To corroborate, we analyzed motility behaviors of Δ*amb0994*, Com*amb0994*, and WT strains. Average swimming velocities of CRISPR-based Δ*amb0994* were significantly faster than those of the complemented strain and WT control (Figure 4D, *P* < 0.05). In addition, compared with those of the control, the diameters and times of “U turn” in the cells of CRISPR-based Δ*amb0994* were smaller and shorter, respectively (Figures 4E,F, *P* < 0.05). These results showed that the “U turn” of CRISPR-based knockout mutant was faster than that of WT cells. The same results were obtained with traditional deletion mutant. The behavior observation, together with CRISPR-based knockdown or knockout mutants, consistently implied that Amb0994 is involved in cellular responses to magnetic torque changes via controlling flagella.

### Amb0994 suppression or deletion reveals a different alpha angle trajectory in “U turn”

**Figure 5:**
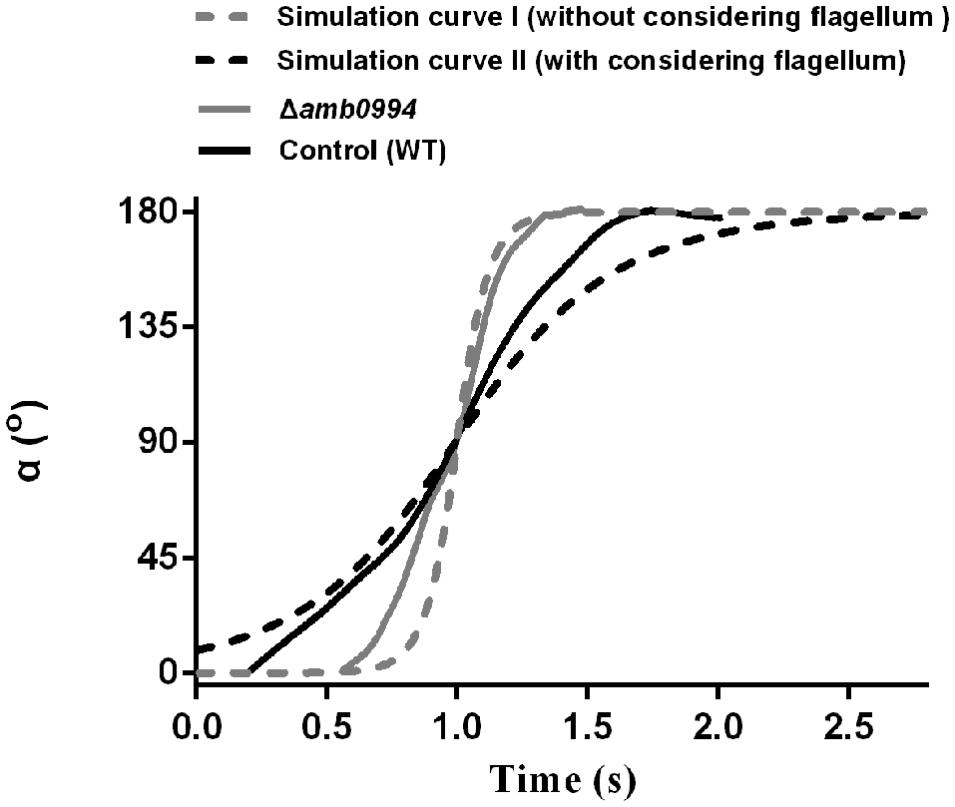
Trajectory of alpha angle after reversing the magnetic field. The black solid line is the trajectory for the WT control cell. The gray solid line is for the CRISPR-based mutant of *amb0994* as Δ*amb0994*. The black dash-line is the simulation curve with considering the effect of the flagellum. The gray dash-line is the simulation curve without considering the flagellum. To compare these curves, we assumed that 90° of the alpha angle occurred simultaneously. The curve is generated from the typical representative data of ~30 bacterial cells.

Previous work found that active sensing exists in magnetotaxis and Amb0994 functions as a magnetic receptor that senses the instantaneous alpha angle (α) between the instantaneous velocity vector and the magnetic field line, which was used to reflect the angle between the cell body and magnetic field direction (Zhu et al., 2014). To study the magnetotactic behavior mechanism, we analyzed the alpha angle trajectory in “U turn” in Δ*amb0994* and control (WT). Our experiments showed that the Δ*amb0994* slop (gray line) was significantly sharper and the time was significantly shorter compared with that (black line) of the control (*P* < 0.05) (Figure 5). This result was consistent with “U turn” data and showed a smaller diameter and less time spent by Δ*amb0994* strain.

To further explain this behavior, we simulated the motion trajectory of bacteria in a magnetic field using an ellipsoidal model with or without considering the flagellar effect. According to the sizes of *M. magneticum* AMB-1 cells, the ellipsoid was assumed as 3.7 μm (major axis diameter) × 0.52 μm (minor axis diameter) × 0.52 μm (minor axis diameter). For modeling *M. magneticum* AMB-1 cells without considering the effect of the flagella, we calculated the resistance factor *C*_*M*_ according to expression of (3) as described in the “Materials and Methods” section. To model *M. magneticum* AMB-1 cells with consideration of the flagella in the mathematical simulation, we chose the experimentally determined resistance factor as 5*C*_*M*_. As shown in Figure 5, the slope without considering the flagella (gray dash-line) is sharper, whereas the time is shorter compared with that (black dash-line) when the flagella is considered. The experimental curve of the Δ*amb0994* is closer to the simulated curve without considering the flagellar effect, whereas the experimental curve of the control is closer to the simulated curve when considering the flagellar effect.

In addition, we analyzed the trajectory for KD*amb0994* and control (no sgRNA). Results showed that the motion curve of KD*amb0994* was closer to the simulation curve without considering the flagella (Figure S2). All the results would suggest that there is a signal, which is transferred to the motors to adjust the flagella and the swimming pattern of the cell when a reversal magnetic field is applied, that indirectly links *amb0994* gene and flagella movement. We further confirmed Amb0994 is essential for magnetic sensing.

## Discussion

Genetic engineering of biological systems possesses significant potential for applications in basic science, medicine, and biotechnology (Ran et al., 2013). Recent work on CRISPR-Cas system has renewed genetic modification (Ran et al., 2013). As an efficient genome editing tool, it can be markedly easy to design and highly specific for gene editing in diverse organisms. Moreover, CRISPRi-dCas9 system has been broadly used in targeting and silencing specific genes without altering the DNA sequence, and it can potentially be adapted for transcriptional regulation on a genome scale in various organisms (Dominguez et al., 2016). Current tools for genome editing have been limited for MTB models. Hence, this is useful to apply the CRISPR-Cas9 system in MTB to improve the efficiency of genome editing.

The conserved MAI of MTB is essential for magnetosome formation, thereby causing the bacterium to passively migrate along the field as it swims (Fukuda et al., 2006). Thus, most of the current studies focus on characterizing the deletion mutants of the MAI genes to analyze the molecular factors and processes in magnetosome biogenesis. The functional analysis of genes that maybe related with an active magnetotactic behavior but not interfere with magnetosome formation is relatively limited. *amb0994* is the gene that is likely to be involved in magnetotactic behaviors, but there is still no direct evidence and even a lack of *amb0994* single gene in-frame deletion mutant to confirm its function. In this study, we first used the CRISPRi technology to specifically repress *amb0994* transcription in attempts to understand its function. We successfully co-expressed dCas9 and sgRNA in *M. magneticum* AMB-1 cells and suppressed *amb0994* expression up to 93% (Figure 1). As the results showed that the dCas9 expression was significantly increased with IPTG induction in these two strains, but dCas9 expression in *M. magneticum* AMB-1 was significantly lower than those in *E. coli*. It could because: (1) Plasmid copy number is different in two strains; (2) different metabolism activity in two host strains, and (3) *M. magneticum* AMB-1 might be more sensitive to dCas9 than *E. coli*. Although CRISPRi can achieve stringent suppression with several sgRNA targeting the same gene, this result might cause off-target effects produced by CRISPRi technology. Thus, to clearly understand the function of *amb0994* gene, we generated a deletion of *amb0994* and its complementation strain to analyze its phenotype in detail. By rationally designing sgRNA, we succeeded in the construction of an in-frame deletion mutant of only *amb0994*. Interestingly, since most bacteria do not have a powerful DNA repair system, the DSB generated by Cas9 is lethal to most microbes (Gomaa et al., 2014; Cobb et al., 2015; Xu et al., 2015). Thus, CRISPR-Cas9 can significantly increase the efficiency of gene engineering in one step, which is an advantage compared with HR procedure since an additional counter-selection is not required. However, the result showed some of the ex-conjugants were mixtures of edited and WT cells in this population. This might be due to the low level of Cas9 in *M. magneticum* AMB-1. Therefore, it is possible to improve Cas9 expression by usage of a codon-optimized gene in the future works. Nevertheless, the observed efficient genome editing occurred. This finding provides a tool for efficiency silencing or deletion of genes in the MTB model strain *M. magneticum* AMB-1. Additionally, to complicate large-scale DNA fragment suppression or deletion, this method will be directed to employ multiple sgRNA targeting different regions to achieve tighter knockdown or knockout of genes to generate editing with high efficiency in MTB (Zeitoun et al., 2015).

The TEM analyses for size, shape factor, number of magnetosomes in one cell, Cmag measurements and cell size were similar to those carrying the sgRNA vector and those that do not. Besides, the synthesis and numbers of flagella were not affected by *amb0994* deletion in amphitrichous bacteria AMB-1. By adding CCCP to interrupt flagellar rotation, we can eliminate the influence of cell motion on Cmag detection. These findings indicated that *amb0994* suppression or deletion in *M. magneticum* AMB-1 do not affect the passive alignment of cells along the magnetic field lines and interfere with the synthesis of magnetosomes. In addition, these results further exclude the concern of potential off-target activity and toxic effect of Cas9 using CRISPR-Cas9 gene editing system. However, in different batches of cultivation strains, factors such as culture conditions and activity of cells might affect the magnetosome formation. Thus, we should choose the same time to cultivate strains with the control and experimental groups to analyze the data in each repeat experiment.

Furthermore, by analyzing the swimming behaviors of *M. magneticum* AMB-1, the swimming velocities of *amb0994* suppression or deletion strains were significantly faster than those of the control. This finding might imply a default basic velocity without regulation of the flagellar motor rotation in the absence of Amb0994 function. In other words, Amb0994 might fine-tune the flagellar rotation speed to adapt to environmental or magnetic disturbances. According to the analysis by Esquivel and De Barros, the diameters of “U turn” in the MTB cells under a reversed magnetic field should be proportional to their swimming velocities, whereas the times of “U turn” should not be related with their swimming velocities (Esquivel and De barros, 1986). However, the experimental results showed that *amb0994* mutants reacted faster compared with control, and the diameters of “U turn” of *amb0994* mutants were smaller than those of the control. By analyzing the dynamics of the *M. magneticum* AMB-1 model, we suggest that the reason for the smaller diameters and faster times of “U turn” of *amb0994* mutants in response to the reversed magnetic field are related to the influence of the flagella. For the wild-type *M. magneticum* AMB-1, the perfect connection between cell body and flagellum causes the cells to have higher resistance in the fluid, which may help withstand perturbations in the magnetic field. For *amb0994* mutants, the absence of Amb0994 function may affect the junction between the flagella and cell body, or decrease the capability of the flagella following the change of the cell body. According to the early model, the magnetosome chains of cells generate magnetic torque when they are not aligned well along the magnetic field lines. The MCP Amb0994 senses the magnetic torque by interacting with MamK, and transfers the signal to the flagellar motors (Philippe and Wu, 2010). However, the motion coordination between the cell body and flagella to respond to magnetic field changes is unclear. Our results suggest that Amb0994 is involved in cellular response to magnetic torque changes via controlling flagella.

Research has shown that cells keep active motility under low magnetic fields, so they would take more time to respond to changes in magnetic field (Lefèvre et al., 2009; Philippe and Wu, 2010). This phenomenon further explains why the cells possessing *amb0994* can be active in sensing the external fields to coordinate their motions, rather than being completely passive to orientation. Another study revealed that *M. magneticum* AMB-1 cells could sense magnetic field gradients and respond to them by reversing direction (González et al., 2015). The author speculates that this difference in the field could be torqueing the cells and relaying this signal to Amb0994. Consequently, combining with theoretical and experimental analyses, we can further confirm the hypothesis that the MCP-like protein Amb0994, as an active magnetic sensor, transmits magnetic signals to the flagellar system to control the movement of the flagella. Bacteria use a widely studied chemotactic signaling pathway to sense various chemicals and physical stimuli, and orient their movements to make them a favorable environment. MCPs are a family of bacterial receptors that detect stimuli and affect the direction of flagella rotation through a signal transduction system (Hazelbauer et al., 2008). These may help bacteria seek favorable habitats quickly in weak geomagnetic fields, which may help achieve better insights on the magnetic response mechanism.

In conclusion, we demonstrated that the type II CRISPR-Cas system of *S. pyogenes* can be reconstituted in *M. magneticum* AMB-1 cells to efficiently and precisely silence or delete genes involved in magnetotaxis. This study provides an efficient and specific genome targeting and editing platform, and paves a new avenue for genetically engineering the magnetotactic model strain. Together the behavior observation and dynamics simulation study indicate that Amb0994 is involved in cellular response to magnetic torque changes via controlling flagella. These imply that the active sensing of magnetic field plays a key role in magnetotaxis.

## Author contributions

HC, TS, and L-FW designed the research and analyzed the data. HC and YC performed the experiments. LC and TS performed dynamics simulation. S-DZ and W-JZ provided the technical support. HC prepared the manuscript. All authors read and approved the final manuscript.

### Conflict of interest statement

The authors declare that the research was conducted in the absence of any commercial or financial relationships that could be construed as a potential conflict of interest.
