# Supplementary material for: Efficient Genome Editing of Magnetospirillum magneticum AMB-1 by CRISPR-Cas9 System for Analyzing Magnetotactic Behavior
Source: Front Microbiol. 2018 Jul 17;9:1569. doi: 10.3389/fmicb.2018.01569 (PMC6056624; doi:10.3389/fmicb.2018.01569)
Supplement: Supplementary file 1 [file Data_Sheet_1.PDF]

Efficient genome editing of *Magnetospirillum magneticum* AMB-1 by CRISPR-Cas9 system for analyzing magnetotactic behavior

Haitao Chen[1,2,3], Sheng-Da Zhang[3,4,†], Linjie Chen[1,2,3], Yao Cai[5], Wei-Jia Zhang[3,4], Tao Song[1,2,3 *], Long-Fei Wu[3,6 *]

[1] Beijing Key Laboratory of Biological Electromagnetism, Institute of Electrical Engineering, Chinese Academy of Sciences, Beijing 100190, China;
[2] University of Chinese Academy of Sciences, Beijing 100049, China;
[3] France-China International Laboratory of Evolution and Development of Magnetotactic Multicellular Organisms, Chinese Academy of Sciences, Beijing 100190, China;
[4] Deep-Sea Microbial Cell Biology, Department of Deep Sea Sciences, Institute of Deep-Sea Science and Engineering, Chinese Academy of Sciences, Sanya 572000, China;
[5] Key Laboratory of Earth and Planetary Physics, Institute of Geology and Geophysics, Chinese Academy of Sciences, Beijing 100029, China;
[6] Aix Marseille Univ, CNRS, LCB, Marseille F-13402, France.

* Correspondence:
Dr. Tao Song
songtao@mail.iee.ac.cn

Dr. Long-Fei Wu
wu@imm.cnrs.fr

† Present address: Sheng-Da Zhang, Department of Biotechnology and Biomedicine, Technical University of Denmark, Kongens Lyngby, 2800, Denmark

Table S1 Plasmids used in this study

Plasmids	Relevant genotype	Reference
PCRISPRi-sgRNA*luxA*	pBBR1-MCS-2 plasmid carrying dcas9 and sgRNA*luxA*, driven by the aTc -inducible tet promoter, Kan^R	(Yin et al. 2018)
PCRISPRi-sgRNA*amb0994*	pBBR1-mcs2 plasmid carrying dcas9 and sgRNA *amb0994*, driven by the IPTG-inducible tac promoter, Kan^R	this study
pUX19	Suicide vector, Kan^R	(Wang et al. 2015a)
pUCGM	Cloning vector, Gm^R	(Wang et al. 2015a)
pUXsuc0994	pUX19 plasmid carrying HDR DNA, Kan^R, Gm^R	this study
pBBR1-mcs2	the broad-host range vector, Kan^R	(Philippe and Wu 2010)
pAK 20	pBBR1-mcs2 plasmid carrying *mamA*-GFP, driven by the IPTG-inducible tac promoter, Kan^R	(Komeili et al. 2006)
pAK0994	pBBR1-mcs2 plasmid carrying *amb0994*-GFP, driven by the IPTG-inducible tac promoter, Kan^R	this study
pCRISPR-*amb0994*	pBBR1-mcs2 plasmid carrying dcas9, sgRNA *amb0994* and HDR DNA, driven by the IPTG-inducible tac promoter, Kan^R, Gm^R	this study

Table S2 Primers used in this study

Primers	Sequence 5′-3′	Reference
sgRNA-*amb0994*-F	ggACTAGTGTAATATCGACCATGATTGGGTTT TAGAGCTAGAAATAGCAAGTTAAAATAAGGC	this study
sgRNA-*amb0994*-R	ccgcTCTAGACAAAAAAAGCACCGACTCGGT GCCACTTTTTCAAGTTGATAACGGACTAGCC TTATTTTAACTTGCTATTTCTAGCTCTAAAAC	this study
sgRNA-Control-F	ggACTAGTGTTTTAGAGCTAGAAATAGCAAG TTAAAATAAGGC	this study
Com *amb0994-EcoRI-gfp*-F	ggcGAATTCATGGAAACGACCCTCGGCTCAT ATG	this study
Com *amb0994-BamHI-gfp*-R	ggcGGATCCCCGCCCGCCAATTCGGGCGATA AA	this study
Com *amb0994-xbaI*-F	gcTCTAGAATGGAAACGACCCTCGG	this study
Com *amb0994-KpnI*-R	ggGGTACCTTACCGCCCGCCAATTC	this study
Cas9-*XhoI*-F1	ccgCTCGAGATGGATAAGAAATACTCAATAG GCTTAGATATC	this study
Overlap Cas9-R2	GAAACTTTGTGGAACAATGTGATCGACATC	this study
Overlap Cas9-F3	GATGTCGATCACATTGTTCCACAAAGTTTC	this study

Cas9-*XhoI*-R4	ccgCTCGAGTTAGTCACCTCCTAGCTG	this study
0994-Left arm-*XbaI*-*XmaI*-F1	gcTCTAGA cccCCCGGGTTCACAAACAGATGCGATAC	this study
0994-Left arm-*BamHI*-R2	cgCGGATCCTTTCCAAAGGCTTCAGCG	this study
0994-Right arm-*BamHI*-F3	cgCGGATCC CTGGTCAGGTTGCTGATG	this study
0994-Right arm-*SacII*-R4	tccCCGCGGATGTCCAATAAGCCAAGTCT	this study
1_0994 -F	CAGGTTCCAGGCATTCA	this study
2_0994-delete-R	AATGGTGACAACTACTG	this study
3_Gm-R	AAGCCTGTTCGGTTCGTA	this study
Gm-F	CGGCGTTGTGACAATTTAC	this study
Check 0995-R	CTTGTCGCCGATCAGAA	this study
P1_*mamC*-F	CTGCGATTCCATCATGCGAAAC	this study
P1_*mamC*-R	CGATCTGATCGCCATCAACGTC	this study
P2_*masA*-F	GCCTGATGGATAGCAACGAAAAAG	this study
P2_*masA* -R	AAAGGACGATAAGGCGCAACAG	this study
P3_*mamB*-F	GTATCCTGGGCTCCAATCTTGTG	this study
P3_*mamB*-R	TGCCGAATACGGCTC AACATAC	this study

P4_*mamY*-F	CGGTTCGGAATGGAATGACCATAG	this study
P4_*mamY*-R	AGCCTTCGGCAAGTTGAATTCC	this study
P5_*mamO′*-F	CCATTCCATCAAGGGACGCTTC	this study
P5_*mamO′*-R	TCTCCTCCACCACGTACAACTG	this study
P6_*mamK*-F	CGAACGGAGTGACAAAAATGAGTG	this study
P6_*mamK*-R	ACCATGCCACTGTCCTAGACTG	this study
Q-*rpoD*-F	ATGGCATCCACCTCAACAAC	(Abreu et al. 2014)
Q-*rpoD*-R	CGTAATAGGCGTCGAGGAAG	(Abreu et al. 2014)
Q-*amb0994*-F	GCGACAGAATTGGAAGCA	this study
Q-*amb0994*-R	CTTGATGGAGGCAGAGAAC	this study
Q-*dCas9*-F	GTCGCCGTTATACTGGTT	this study
Q-*dCas9*-R	GTCCAGACACTTGTGCTT	this study

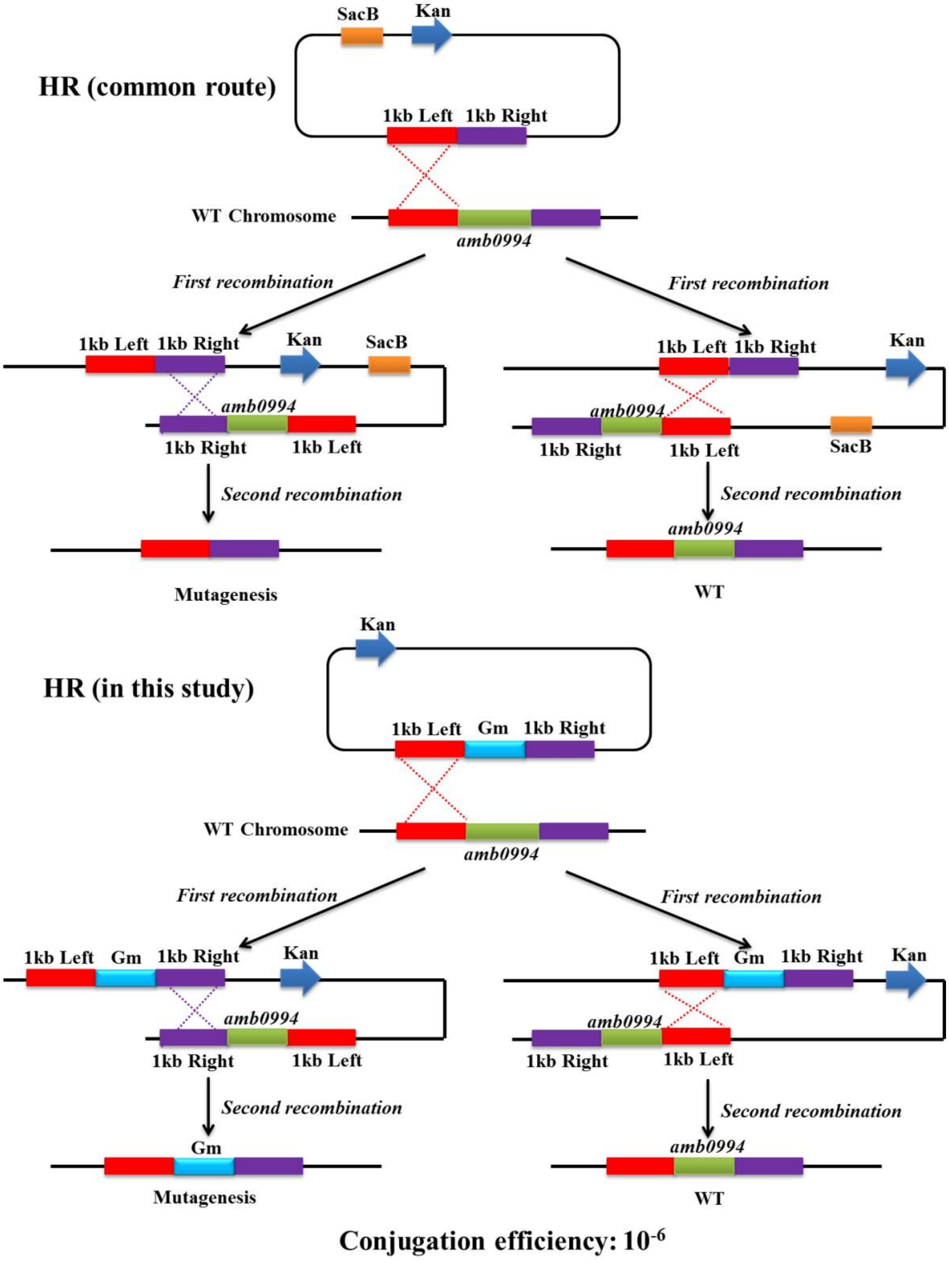

Figure S1 Schematic presentation of mechanism for HR.

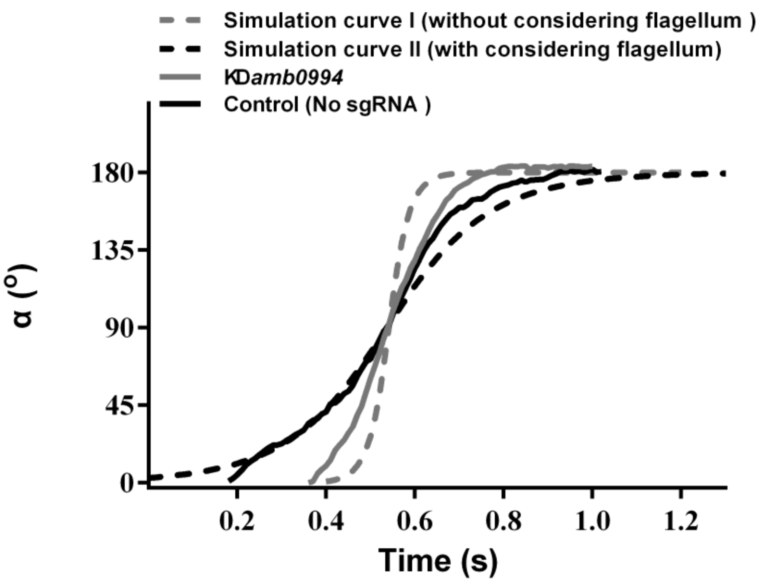

Figure S2 Trajectory of alpha angle after the magnetic field is reversed in KD*amb0994* and control (no sgRNA) cell.

Movie S1 This movie shows the trajectory of “U turn” after the magnetic field is reversed. The swimming of *M. magneticum* AMB-1 strains control (no sgRNA) was recorded at 33 fps with a 40× objective. The magnetic field was 1 mT in the “U turn” experiment without shielding the geomagnetic field. Red and blue arrows represent the direction of the magnetic field.

Movie S2 This movie shows the swimming behaviors of KD*amb0994* in the response to a magnetic field.
